# Reply to “Chronic Vaginal Candidiasis Is Achievable in Outbred CD-1 Mice”

**DOI:** 10.1128/mBio.01736-17

**Published:** 2017-10-24

**Authors:** Junko Yano, Mairi C. Noverr, Paul L. Fidel

**Affiliations:** Louisiana State University Health Sciences Center, Center of Excellence in Oral and Craniofacial Biology, New Orleans, Louisiana, USA; Albert Einstein College of Medicine

**Keywords:** *Candida albicans*, inflammation, polymorphonuclear neutrophils, vaginitis

## REPLY

We appreciate the attention given to our publication regarding the mechanism associated with the immunopathogenic response in experimental vulvovaginal candidiasis that employed mice resistant (CD-1) and susceptible (C3H/HeN) to chronic colonization/infection (6). We respect data presented by Gabrielli et al. (1) regarding the achievement of a chronic colonization condition in the CD-1 strain of mice that were characterized as resistant in our report (6). As an experimental model system, the conditions can often be pushed in one direction or another that may produce variable phenotypes. We believe that these contrary observations are a classic example of employing conditions that altered the phenotype. Our laboratory published data for the vaginal infection using inocula of 5 × 10^4^ to 5 × 10^5^ blastoconidia and exogenous estrogen between 0.2 and 0.5 mg/mouse/week for several years based on early optimization studies. We then conducted a lower-limit analysis for experimental vaginitis using mice susceptible to long-term colonization that included a dose response of inocula (1 × 10^2^ to 5 × 10^4^ blastoconidia) as well as exogenous estrogen (0.002 to 0.2 mg/week) (2). On the basis of these studies, 5 × 10^4^ blastoconidia were found to be an optimal inoculum based on the fact that lower inocula (1 × 10^2^ to 1 × 10^3^) resulted in variability in fungal burden. In addition, the 5 × 10^4^ inoculum reproducibly resulted in stable fungal burden at levels similar to those observed at higher inocula (1 × 10^6^ to 1 × 10^7^). The estrogen dose was chosen in a similar manner (0.02 mg/week). Interestingly, the estrogen dose was ultimately increased to 0.1 mg/week, as random variability in fungal burden was observed when different lots of estrogen were used. The inoculum used by Gabrielli et al. (1) was 40× higher (2 × 10^6^) than the inoculum we optimized for the susceptible mice and even higher (400× at 2 × 10^7^) in their previous published work (3). Therefore, it is not surprising that the resistant phenotype was overcome, resulting in a conversion toward a susceptible phenotype.

To examine the inoculum issue further, we conducted a study that evaluated the effect of inocula in susceptible (C3H/HeN) mice on the outcome of the acute inflammatory response via polymorphonuclear neutrophil (PMN) migration and ultimately, vaginal fungal burden. Our goal was to determine whether the susceptible phenotype could be similarly converted to a resistant phenotype with lower inocula. For this, the standard inoculum (5 × 10^4^), lower-limit inoculum (1 × 10^2^), and low inoculum (5 × 10^2^) of *Candida albicans* 3153A was used. Note that the lower-limit and low inocula were 500-fold and 100-fold, respectively, below the standard inocula. Longitudinal analysis of PMN migration and vaginal fungal burden was monitored for 15 days. Results showed that mice receiving the lower-limit inoculum did not become colonized and did not show PMN migration to the vaginal cavity. Hence, the lower-limit inoculum in the susceptible mice did not show a resistant phenotype with a clearing PMN response. Instead, it appeared that the inoculum was too low to support colonization to trigger a PMN response. Mice receiving the low and standard inocula both showed the characteristic high-level fungal burden (>10^4^ CFU/100-µl lavage fluid sample) over the 15 days, together with a prominent nonclearing PMN response. Hence, even a near-lower-limit inoculum failed to show a resistant phenotype. Thus, it does not appear that susceptible mice can be converted to a resistant phenotype by altering the inocula.

Another major issue is the inherent differences in estrogen responsiveness in the mouse strains. The pseudoestrus state is required to achieve consistent colonization following inoculation. This is consistent with a major risk factor for vaginitis in women (high estrogen levels during menstrual cycle and/or high-estrogen oral contraceptives/hormone replacement therapy). All the chronic vulvovaginal candidiasis (CVVC)-susceptible mouse strains mentioned have normal responsiveness to estrogen. CD-1 mice, on the other hand, have been shown to be significantly less responsive to exogenous estrogen (4). This was presumably the primary factor in the inability of the mice to consistently maintain fungal burden following inoculation (5). Our studies, for the first time however, showed an appreciable PMN response in inoculated CD-1 mice treated with exogenous estrogen or not treated without exogenous estrogen, concomitant with reduced fungal burden. Subsequent PMN depletion studies indicated that the loss of fungal burden was mediated by the infiltrating PMNs. Gabrielli et al. do in fact treat the CD-1 mice with a substantial dose of estrogen (0.2 mg/mouse) (1).

Taking all data together, we contend that there are distinct susceptible and resistant mouse strain phenotypes for experimental vaginitis at the standard inoculum used for susceptible mice. This is supported by the mechanism uncovered for the susceptible phenotype involving heparan sulfate (HS) that acts as a competitive inhibitor of the Mac-1/Pra1 interaction between the neutrophil and *Candida* that inhibits the PMN-mediated antifungal activity. CD-1 mice lack sufficient HS and effectively clear the infection/colonization via infiltrating PMNs when inoculated with the standard inocula. However, as this is an experimental model system, we recognize that this resistant phenotype in CD-1 mice may be variable when challenged with higher inocula.
